# Advances in chemical analysis of micro- and nanoplastics

**DOI:** 10.1007/s00216-023-04747-y

**Published:** 2023-05-26

**Authors:** Natalia P. Ivleva, Sebastian Primpke, Jennifer M. Lynch

**Affiliations:** 1 Institute of Water Chemistry, Chair of Analytical Chemistry and Water Chemistry, Technical University of Munich, Lichtenbergstr. 4, 85748 Garching, Germany; 2 Alfred-Wegener-Institute Helmholtz Centre for Polar and Marine Research, Biologische Anstalt Helgoland, Kurpromenade 201, 27498 Helgoland, Germany; 3 National Institute of Standards and Technology, Chemical Sciences Division, Waimanalo, HI 96795 USA; 4 Hawaii Pacific University, Center for Marine Debris Research, Waimanalo, HI 96795 USA

Microplastics and nanoplastics are tiny fragments of synthetic polymers (plastics), found in the environment (including sea- and fresh water, sediments, biota, soils, and air) as well as in drinking water and food, and therefore recognized as emerging particulate anthropogenic contaminants. Plastic particles, including fibers, in the size range of 5 mm to 1 mm are referred to as large microplastics, from 1 mm to 1 μm are defined as microplastics, and smaller than 1 μm are nanoplastics (ISO/TT 21960). The numerous reports on the occurrence of microplastics and, recently, nanoplastics raised concerns about their impacts on the environment and human health. Generally, more hazardous effects are expected from smaller microplastics and, especially, from nanoplastics. In order to adequately and systematically address these issues, reliable information on the ambient concentrations of microplastics and nanoplastics are required.

The diversity and complexity of plastic sources, material properties, usage patterns, and emission pathways are reflected in the diversity of microplastic and nanoplastic particles found in the environment. These particles exhibit a large variety of chemical and physical characteristics (e.g., polymer type, size, shape, density, surface properties, additives, extent of weathering, etc.). Therefore, advanced methods are required for the reliable analysis of microplastics and nanoplastics, and they are probably some of the most challenging analytes to measure in environmental samples. The analysis must include representative sampling and efficient sample preparation, as well as chemical identification, quantification, and characterization of the particles in the entire size range and in different media. All together, these challenges make it necessary to optimize existing and develop and validate new efficient methods. Time- and cost-efficiency of methods must be considered, and high throughput analysis is ideal for monitoring studies. Furthermore, methods providing detailed information on the chemical composition, particle number, size distribution, shape, surface properties, associated additives, and sorbed contaminants, weathering state, etc., are needed for better understanding of the micro- and nanoplastic fate in the environment and for thorough assessment of the potential eco-toxicological risks.

Comparison, harmonization, and standardization within the same/similar methods (e.g., mass-based and/or particle-based) as well as between principally different, but complementary approaches, are greatly needed. However, the availability of suitable reference materials for microplastics and nanoplastics which mimic real analytes, including different polymer types, broad size range, and different shapes is still limited, hampering the development of harmonized and standardized methods for the analysis of microplastics. Methods for the identification and quantification of nanoplastics are still under development, and their comparison and harmonization will be a topic of future studies.

Therefore, we organized this Topical Paper Collection “*Advances in Chemical Analysis of Micro- and Nanoplastics*” and invited worldwide experts working in this field to highlight the development, optimization, validation, application, and harmonization of advanced methods for the analysis of microplastics and nanoplastics in different matrices.

This topical paper collection includes 12 research articles and one critical review. The research articles cover all steps of micro- and nanoplastic analysis, including sampling, sample preparation, polymer identification, quantification, and characterization. Here, mass-based approaches (pyrolysis-gas chromatography/mass spectrometry (py-GC/MS) and proton nuclear magnetic resonance (^1^H-NMR)) are further developed, optimized, and applied for the analysis of micro- and nanoplastics in environmental samples, as well as for the characterization of test/reference materials. As particle-based approaches, vibrational spectroscopy methods are strongly presented, including μ-Raman, μ-FTIR spectroscopy and NIR hyperspectral imaging enabling the reliable analysis of microplastics down to approx. 1 μm (for detailed analysis), 10 μm and 100 μm to 300 μm (for high-throughput monitoring), respectively. Furthermore, approaches for the physicochemical analysis of nanoplastics are presented and critically evaluated. Finally, essential topics on the production of reference materials for microplastics, their characterization and application for interlaboratory comparison exercises are discussed.

We sincerely thank all authors for contributing their valuable work and the reviewers for critically assessing and improving the papers for this topical collection “*Advances in Chemical Analysis of Micro- and Nanoplastics*”. We are also very grateful to the editorial office for the efforts in helping create and compile this collection. We are proud of the quality of contributions and strongly believe that this topical collection will be of great interest to researchers not only in the interdisciplinary field of microplastics and nanoplastics, but also far beyond.

